# Multi-Method Integration for Spectral Band Importance Analysis in Coal Characterization

**DOI:** 10.3390/s25237155

**Published:** 2025-11-24

**Authors:** Jie Zhang, Tianju Zhao, Youquan Dou, Zhiyuan Liu, Yu Zhou

**Affiliations:** 1 National Environmental Protection Research Institute for Electric Power Co., Ltd., Nanjing 210031, China; 12051884@ceic.com (J.Z.); 12050113@ceic.com (Y.D.); 2 Guoneng Nanjing Coal Quality Supervision and Inspection Co., Ltd., Nanjing 211800, China; 3 School of Information and Control Engineering, China University of Mining and Technology, Xuzhou 221116, China; tjzhao@cumt.edu.cn; 4 School of Software, Dalian University of Technology, Dalian 116620, China; 20202241324@mail.dlut.edu.cn

**Keywords:** near-infrared spectroscopy, multi-method analysis, coal quality assessment, moisture in air-dried basis (*M_ad_*), volatile matter in air-dried basis (*V_ad_*), machine learning

## Abstract

To improve the accuracy and reliability of coal quality assessment via near-infrared spectroscopy, this study proposes a multi-method analysis framework for robust spectral feature selection. A core challenge is reconciling the trade-offs between different analytical approaches: statistical methods often yield smooth but diffuse results, while machine learning models can identify sharp, localized features that may lack stability. Our framework addresses this by integrating diverse analytical perspectives, including statistical correlations, SHAP-interpreted machine learning models, and latent-variable regression. We then introduce a novel fusion strategy that synthesizes the importance profiles from these methods based on inter-method consistency, curve smoothness, and local concentration. Experimental results demonstrate this fusion yields more interpretable and physicochemically coherent wavelength importance profiles for both Moisture ($M_{ad}$) and Volatile Matter ($V_{ad}$). The selected features consistently achieve superior prediction performance across various regression models, showing particular robustness with limited training data. This work offers a structured methodology for identifying compact and informative spectral features, facilitating the development of efficient models for online monitoring and contributing to improved process control.

## 1. Introduction

Coal remains one of the most important energy sources worldwide, and its physicochemical properties directly influence combustion efficiency, storage safety, and the economic feasibility of industrial applications. Rapid and accurate evaluation of key parameters such as moisture and volatile matter is therefore essential for optimizing combustion processes and mitigating environmental impacts. Traditionally, proximate analysis of coal (moisture, ash, volatile matter, and fixed carbon) follows standardized procedures [1]. Although reliable, these gravimetric methods are time-consuming, involve complex sample handling, and are unsuitable for real-time, on-line monitoring. To address these challenges, both academia and industry have pursued rapid, non-destructive detection technologies. Spectroscopic approaches are particularly attractive because they are efficient, reagent-free, and non-destructive. In addition to near-infrared (NIR) spectroscopy, laser-induced breakdown spectroscopy (LIBS) for elemental analysis [2] and microwave spectroscopy for moisture determination [3] have shown considerable potential.

Among these, NIR spectroscopy was selected due to its direct mechanistic link to the target properties. NIR is highly sensitive to the O-H and C-H molecular vibrations characteristic of moisture and volatile matter, providing a robust analytical foundation [4]. In contrast, alternative methods like Raman spectroscopy are severely hampered by fluorescence interference from the coal matrix, while Terahertz spectroscopy is often impractical for industrial settings due to high costs and environmental sensitivity. NIR thus offers the most effective and feasible solution for this application. NIR measurements, when combined with machine learning models, enable efficient, large-scale prediction of coal quality while reducing analysis time and cost [5]. Recent studies have leveraged deep learning to process spectral data; for example, multi-output networks can perform intelligent, simultaneous proximate analysis from NIR spectra [6]. To achieve robust prediction, it is essential to consider how spectral information is preprocessed, transformed, and summarized into informative features. Effective feature extraction addresses common NIR challenges such as noise and baseline drift, scattering effects, high dimensionality, collinearity among wavelengths, and nonlinear relationships between spectral signatures and target properties. Carefully designed features not only enhance model accuracy and robustness but also improve interpretability by highlighting the spectral regions most relevant to coal quality.

Current feature-extraction approaches generally include preprocessing, dimensionality reduction, variable selection, and information-based measures. However, most studies tend to employ a **single analytical method** to establish the relationship between spectral features and coal properties. This practice risks overlooking complementary perspectives from other approaches and may lead to unstable or biased conclusions. In related spectral domains (e.g., microwave spectroscopy), deep learning-based adaptive fusion of multi-source spectral information has been shown to significantly improve detection performance, reinforcing the limitations of single-method strategies and the trend toward multi-method, multi-information integration [7].

To overcome this limitation, the present study proposes a **multi-method analysis framework**. This approach is conceptually inspired by recent breakthroughs in multi-modal learning, where fusing information from disparate data types such as images and text has led to significant gains in model performance and robustness [8,9,10,11]. Within our framework, multiple families of feature-extraction methods are first applied independently. Correlation-based measures (e.g., Pearson [12], Spearman, distance correlation [13], and Hilbert–Schmidt independence criterion [14]) emphasize global associations and produce smooth and stable importance profiles, but their reliance on global correlation can lead to overly diffuse signals and loss of local discriminative detail. In contrast, machine learning models (e.g., random forest [15], XGBoost [16], LightGBM [17], and CatBoost [18] combined with SHAP [19] interpretation) can capture sharp, localized discriminative regions, but their outputs are often volatile and lack reproducibility across adjacent wavelengths. By bringing together methods with complementary characteristics, the framework is designed to achieve both smoothness and resolution, avoiding the trade-offs that arise when only one methodological family is used.

Building on this diverse foundation, the framework further introduces a structured fusion stage. Here, we first derive a consensus profile across methods, then evaluate the contribution of each approach relative to this consensus. To account for family-specific biases, we adjust the weights of individual methods using smoothness and local concentration criteria, thereby attenuating the volatility of model-driven approaches and the diffuseness of correlation-based ones. The resulting fused profiles preserve global trends while retaining localized discriminative information.

In this way, the proposed framework constitutes a holistic innovation: it integrates a carefully selected set of complementary single-modality analyses and unifies them through an adaptive fusion mechanism. This dual design not only ensures systematic comparison of diverse methods but also yields a robust and interpretable basis for feature selection in spectroscopic modeling, offering a generalizable paradigm for exploring the relationship between spectral signatures and material properties.

### 1.1. Data Description

The dataset used in this study comprises two key coal property indicators: Volatile Matter in Air-Dried Basis ($V_{ad}$) and Moisture in Air-Dried Basis ($M_{ad}$). $V_{ad}$ refers to the content of volatile matter in coal measured under air-dried conditions, which is closely related to coal’s combustibility and gas generation rate. $M_{ad}$ represents the inherent moisture content, a critical parameter for determining combustion efficiency and storage stability. While other indicators (e.g., ash content, calorific value) also reflect coal quality, this study focuses on $M_{ad}$ and $V_{ad}$ because their strong correlation with the vibrational characteristics of organic functional groups makes them highly suitable for NIR analysis.

In total, 739 samples were collected. The samples were sourced from multiple batches of raw coal imported through a major port, covering a diverse range of geographical origins and coal types. This diverse sourcing ensures significant variability in coal quality, making the dataset highly representative for practical applications such as raw coal trading, import inspection, and rapid quality assessment, thereby supporting the model’s generalizability to real-world industrial scenarios.

The spectral data were collected from 908.1 nm to 1676.2 nm. This operational range, a characteristic of the portable spectrometer used, is particularly well-suited for coal analysis as it covers the primary absorption bands (e.g., −OH, −CH, $-{\mathrm{CH}}_{2}$, and $-{\mathrm{CH}}_{3}$) relevant to moisture and volatile matter. The spectral interval of 6.19 nm is an inherent resolution parameter of the instrument, sufficient for resolving the broad characteristic peaks of coal while ensuring the rapid and stable signal collection necessary for portable applications.

**Figure 1:**
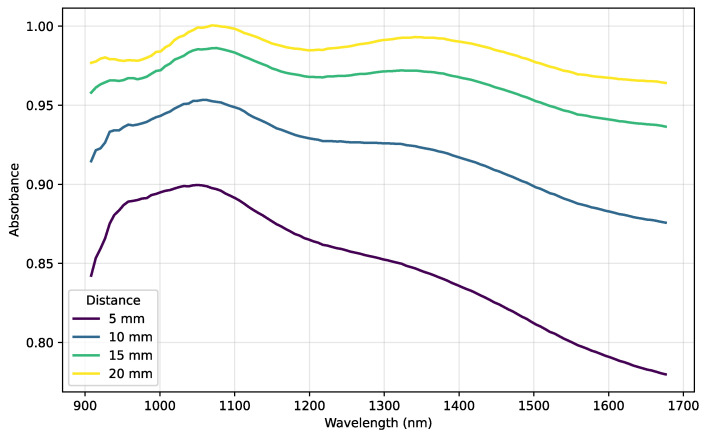
Effect of sampling distance on the NIR spectrum of a single coal sample. Variations in absorbance and baseline shifts underscore the importance of maintaining consistent measurement conditions.

All samples were obtained under strictly consistent measurement conditions to ensure comparability and reliability. This is critical, as variations like sampling distance can introduce significant spectral shifts. To illustrate this, Figure 1 displays the NIR spectra of the same coal sample measured at different distances. The resulting spectral variations clearly arise from inconsistent sampling, not intrinsic sample properties, underscoring the necessity of a fixed measurement protocol for reliable data acquisition.

**Figure 2:**
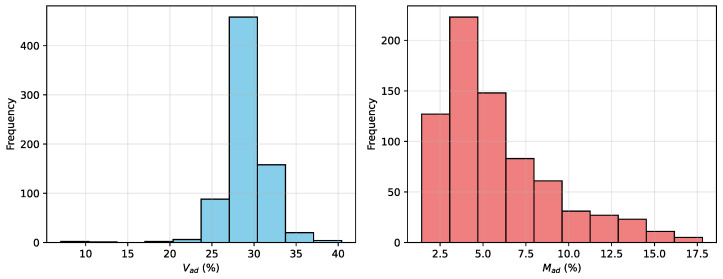
Distributions of $V_{ad}$ and $M_{ad}$, showing concentrated spread for $V_{ad}$ and relatively dispersed spread for $M_{ad}$.

Figure 2 presents the statistical distributions of $V_{ad}$ and $M_{ad}$ across the dataset. The mean value of $V_{ad}$ is 29.08 with a standard deviation of 2.61, while $M_{ad}$ has a mean of 5.86 and a standard deviation of 3.25. Both histograms exhibit relatively concentrated distributions.

**Figure 3:**
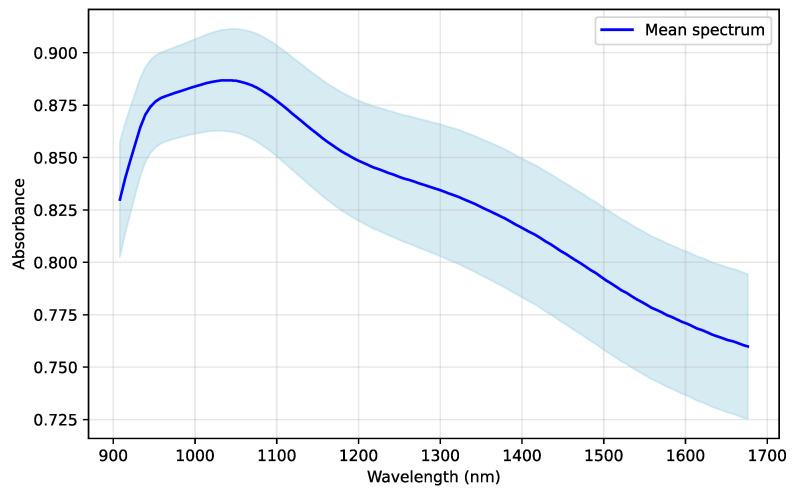
Mean and standard deviation curves of NIR spectra (908.1–1676.2 nm).

Figure 3 illustrates the mean and standard deviation of the collected NIR spectra. The solid curve denotes the mean absorbance and the shaded region indicates one standard deviation. The results show that absorbance remains relatively stable across the spectral range, with mean values between 0.83 and 0.88.

**Figure 4:**
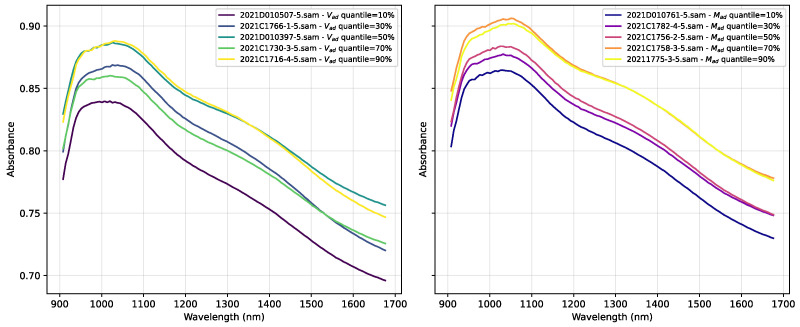
NIR spectra of representative samples at different quantiles for $V_{ad}$ (**left**) and $M_{ad}$ (**right**).

To further investigate the relationship between chemical properties and spectral features, we selected representative samples based on the distributions of $M_{ad}$ and $V_{ad}$. Specifically, samples corresponding to the 10%, 30%, 50%, 70%, and 90% quantiles of each property were chosen. The NIR spectra for these representative samples are plotted in Figure 4. The figure clearly demonstrates that as the values of $V_{ad}$ (left panel) and $M_{ad}$ (right panel) systematically change, the corresponding spectral absorption peak positions and intensities also exhibit regular variations. This provides strong evidence of significant chemical composition differences within the dataset, and confirms that these differences are effectively captured by the NIR spectra.

### 1.2. Data Preprocessing

To ensure the comparability and consistency of multi-method data during the modeling process, the raw NIR spectral dataset was first subjected to completeness screening. Since correlation analysis between different data sources (i.e., physicochemical indicators and spectral information), any sample with missing values in either modality was excluded. This procedure yielded a unified dataset with complete physicochemical parameters and spectral features, thereby providing a consistent foundation for subsequent modeling.

The spectral data were then smoothed using a Savitzky–Golay filter to reduce high-frequency noise introduced during measurement, while preserving peak shapes and improving the signal-to-noise ratio [20]. To further minimize the influence of physical factors such as particle size and surface scattering, standard normal variate (SNV) transformation was applied to standardize the spectral intensity distribution of each individual sample [21]. In addition, multiplicative scatter correction (MSC) was implemented with a reference spectrum to eliminate global offsets and slope variations across samples caused by scattering effects [22,23]. These preprocessing steps effectively enhanced the correlation between spectral data and coal property indicators, thereby improving the robustness of feature selection and interpretability analysis.

## 2. Methodological Framework

**Figure 5:**
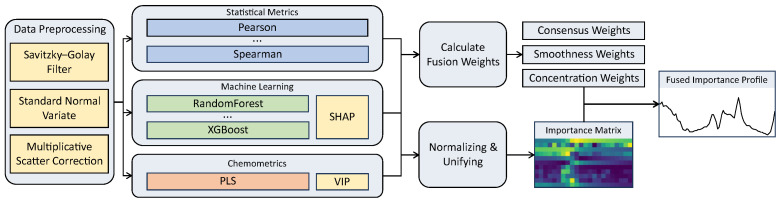
Architectural diagram of the proposed multi-method analysis framework.

Our work introduces a multi-method framework to systematically integrate diverse analytical perspectives. The architecture of this framework is illustrated in Figure 5. The framework operates in two main stages. First, a parallel single-modality analysis is performed, where a diverse set of methods independently generates importance profiles from the preprocessed spectral data. Second, a multi-method integration stage adaptively fuses these individual profiles. The fusion process is guided by three quantitative metrics: inter-method consistency, smoothness, and local concentration. These metrics determine the final weight assigned to each method, and the final output is a single, robust feature importance profile created through a weighted aggregation.

The core innovation of this framework is its systematic integration of multiple, methodologically diverse approaches, rather than a reliance on any single technique. We recognize that each family of methods offers a distinct perspective with inherent trade-offs. For example, statistical correlation-based methods tend to provide smooth, global insights but may sacrifice the resolution of local details. In contrast, machine learning models can identify sharp, localized features, but their results are often prone to instability. Our framework treats these different analytical techniques as complementary “modalities.” It is designed to fuse their respective strengths and mitigate the limitations of any single approach. The following subsections will first detail the individual single-modality analyses and then describe the fusion strategy used to produce a unified, robust, and physically interpretable feature importance profile.

### 2.1. Component Analytical Methods

In characterizing the relationship between spectral features and coal properties, different analytical approaches embody distinct perspectives. Linear and conditional linear correlation methods (e.g., Pearson correlation [12], partial correlation) capture linear dependencies through concise parametric formulations. While the interpretations are straightforward, these methods are limited in their ability to detect nonlinear structures. The monotonic correlation method (e.g., Spearman correlation) employs rank-based statistics to assess monotonic, potentially nonlinear relationships. This approach is advantageous due to its scale invariance and robustness against outliers. General nonlinear dependence measures (e.g., mutual information [24], distance correlation [13], Hilbert–Schmidt independence criterion [14]) go beyond linear and monotonic assumptions, offering a comprehensive characterization of arbitrary dependency structures within the frameworks of information theory, energy statistics, and kernel methods. The predictive modeling and interpretability approach (e.g., Random Forest [15], XGBoost [16], LightGBM [17], CatBoost [18] combined with SHAP analysis [19]) leverages the strong fitting capabilities of machine learning models to capture complex, high-dimensional relationships. With SHAP values, these models further enable interpretability from local to global scales. The latent-variable regression approach (e.g., PLS with Variable Importance in Projection, VIP) integrates dimensionality reduction and regression, providing stable importance estimation under severe spectral collinearity while adhering to the chemometric tradition.

Together, these methods highlight complementary perspectives on the spectrum– property relationship, ranging from simple correlation-based measures to advanced machine learning and chemometric modeling. In the following subsections, each method is introduced in detail, with an emphasis on its methodological principles, strengths, and limitations in the context of coal spectral analysis.

#### 2.1.1. Pearson Correlation Coefficient

The Pearson correlation coefficient is a widely used metric that quantifies the strength and direction of a linear association between two variables [12]. In spectral analysis, it offers a straightforward assessment of the linear relationship between a single wavelength and the target property, but it is unable to capture nonlinear dependencies.

#### 2.1.2. Partial Correlation

Partial correlation measures the linear association between a given wavelength and the target variable after controlling for the confounding effects of other wavelengths. By removing the influence of multicollinearity, this approach provides a more accurate characterization of the independent contribution of a single spectral feature.

#### 2.1.3. Spearman’s Rank Correlation Coefficient

Spearman’s rank correlation is a non-parametric measure that evaluates the monotonic relationship between two variables. Because it operates on ranks rather than raw values, it is robust to outliers and does not assume a linear relationship, making it well-suited for spectral data that often exhibit nonlinear effects.

#### 2.1.4. Mutual Information (MI)

Derived from information theory, Mutual Information (MI) quantifies the statistical dependence between two variables by measuring the amount of information shared between them [24]. A larger MI value indicates stronger dependence. As a generalized measure, MI can effectively identify complex nonlinear associations, making it a powerful tool for feature selection beyond linear constraints.

#### 2.1.5. Distance Correlation (dCor)

Distance Correlation (dCor) is a powerful statistical measure capable of detecting both linear and nonlinear dependencies [13]. Critically, its value is zero if and only if the variables are statistically independent. This property makes dCor particularly effective in uncovering arbitrary dependence structures within spectroscopic data.

#### 2.1.6. Hilbert–Schmidt Independence Criterion (HSIC)

The Hilbert–Schmidt Independence Criterion (HSIC) is a kernel-based measure used to test for statistical independence between variables in a Reproducing Kernel Hilbert Space (RKHS) [14]. By mapping data into a high-dimensional feature space, HSIC can capture complex and subtle relationships, offering unique advantages for feature selection in high-dimensional spectral analysis.

#### 2.1.7. Random Forest

Random Forest is an ensemble method that aggregates multiple decision trees using bagging to reduce variance and capture nonlinear patterns [15]. While traditional importance metrics based on impurity reduction can be biased toward features with high cardinality, this study employs SHAP (SHapley Additive exPlanations) [19] to interpret the model. SHAP decomposes predictions into marginal contributions based on cooperative game theory, providing a consistent and unified measure of wavelength importance that balances interpretability with the model’s strong predictive capability.

#### 2.1.8. XGBoost

XGBoost (eXtreme Gradient Boosting) improves upon basic boosting frameworks through efficient implementation and regularization terms that effectively control overfitting [16]. Its ability to model complex, high-order nonlinear spectral relationships makes it highly suitable for this task. By integrating XGBoost with SHAP, we move beyond standard node-split statistics to obtain an unbiased, attribution-based assessment of each wavelength’s contribution to the coal property prediction.

#### 2.1.9. LightGBM

LightGBM (Light Gradient Boosting Machine) utilizes histogram-based splitting and leaf-wise growth strategies, offering superior training efficiency particularly for high-dimensional data [17]. In our framework, the SHAP explainer is applied to interpret LightGBM outputs. This approach accurately quantifies the marginal contribution of wavelengths, avoiding the potential inconsistencies found in built-in gain or split-count metrics, and ensuring robust feature selection even with large-scale spectral features.

#### 2.1.10. CatBoost

CatBoost distinguishes itself with ordered boosting and advanced processing of feature combinations, effectively mitigating prediction bias and overfitting [18]. Although spectral data are continuous, CatBoost’s robust regularization mechanisms make it a strong candidate for stable modeling. SHAP analysis is applied here to provide a consistent importance distribution, ensuring that the interpretation remains comparable across different tree-based models within our framework.

#### 2.1.11. Partial Least Squares (PLS) Regression + VIP Scores

Partial Least Squares (PLS) regression is a cornerstone chemometric approach designed to handle severe multicollinearity in spectral data [25]. It projects predictors into a latent space to maximize covariance with the response variable. To assess the contribution of original wavelengths, we employ the Variable Importance in Projection (VIP) score. Wavelengths with VIP scores greater than 1 are generally considered significant, providing a stable and quantitative criterion for feature selection rooted in the chemometric tradition.

Taken together, these methods emphasize different perspectives: some prioritize simplicity and linearity, others pursue universal measures of nonlinear dependence, and machine learning models offer high-dimensional predictive power. This methodological diversity lays the foundation for the multi-method integration. By treating these approaches as complementary components and synthesizing their results, we can exploit their individual strengths while mitigating the biases of any single method, ultimately yielding a more comprehensive, robust, and interpretable conclusion regarding spectral band importance.

### 2.2. Multi-Method Integration Approaches

In spectroscopic modeling research, different variable selection and importance evaluation methods often yield divergent results. To fully exploit the complementarity of these approaches, this study proposes an ensemble strategy based on multi-metric evaluation, which integrates multiple importance estimates into a unified composite curve. The core of the method lies in establishing a well-defined evaluation framework and determining the integrated weights for different methods accordingly. Specifically, the design and assessment are carried out from three perspectives: inter-method consistency, curve smoothness, and local segment concentration.

**Inter-method Consistency Metric:**

To quantify the consistency between the results of different methods and the overall trend, this study employs Non-negative Matrix Factorization (NMF) to construct a consensus pattern. Let the original importance matrix be

(1) $$X\in R^{n\times m},\;X\geq 0$$

where *n* denotes the number of wavelengths and *m* denotes the number of methods. By applying NMF, *X* can be decomposed as

(2) $$X\approx WH,\;W\in R^{n\times r},H\in R^{r\times m},\;W,H\geq 0$$

When r=1, *W* can be regarded as the global consensus curve, while *H* represents the contribution coefficients of each method to this consensus. After normalization, the weights derived from *H* are denoted as $w_{j}^{\left(1\right)}$, which serve as the inter-method consistency metric, reflecting the degree to which method *j* aligns with the overall pattern.

**Smoothness Metric:**

Spectral signals typically exhibit continuity, and the distribution of importance across wavelengths should not fluctuate excessively. To address this, we introduce a smoothness metric based on Total Variation (TV). Let the importance curve obtained by a given method be

(3) $$c=\left(c_{1},c_{2},\ldots ,c_{n}\right)$$

The total variation of *c* is defined as

(4) $$TV\left(c\right)=\sum_{i=1}^{n-1}\left|c_{i+1}-c_{i}\right|$$

Accordingly, the smoothness score is defined as

(5) $$S\left(c\right)=\frac{1}{1+TV(c)}$$

A higher smoothness score indicates a more gradual variation in the curve, which better conforms to the physical characteristics of spectral variables. After normalization, the smoothness metric is denoted as $w_{j}^{\left(2\right)}$.

**Local Segment Concentration Metric:**

The characteristic information of spectral signals is often concentrated within locally continuous intervals. To capture this property, we propose a local segment concentration metric to quantify the degree of energy concentration within finite wavelength ranges. Let the total curve energy be defined as

(6) $$E_{\mathrm{total}}=\sum_{i=1}^{n}c_{i}^{2}$$

and denote the window length by *L*. For all sliding windows of length *L*, the local energy is computed as

(7) $$E_{k}=\sum_{i=k}^{k+L-1}c_{i}^{2}$$

The segment concentration score is then defined as

(8) $$R\left(c\right)=\max_{k}\frac{E_{k}}{E_{\mathrm{total}\;}}.$$

A higher value of this metric indicates that the contribution is more concentrated within a specific continuous interval, which better reflects the mechanistic characteristics of spectral variables. After normalization, the local segment concentration metric is denoted as $w_{j}^{\left(3\right)}$.

First, we calculate three key metrics: inter-method consistency, smoothness, and local segment concentration. To ensure these metrics are comparable, each one is normalized. We then integrate them using a dynamic weighting strategy, where the weight for each metric is determined by its discriminative power. This power is quantified by the standard deviation of its scores across all methods, which is then transformed into a stable weight using a temperature-controlled softmax function and a convex combination with a uniform distribution.

Let the dynamically determined weights for inter-method consistency, smoothness, and local segment concentration be $\omega^{\left(1\right)},\omega^{\left(2\right)},$ and $\omega^{\left(3\right)}$, respectively. The final weight for method *j* is defined as:

(9) $$W_{j}=\omega^{\left(1\right)}w_{j}^{\left(1\right)}+\omega^{\left(2\right)}w_{j}^{\left(2\right)}+\omega^{\left(3\right)}w_{j}^{\left(3\right)}$$

which is further normalized to satisfy $\sum_{j}W_{j}=1$. Finally, the importance curves of all methods are aggregated through weighted summation to yield the fused curve:

(10) $$C_{\mathrm{fused}}\left(i\right)=\sum_{j=1}^{m}W_{j}c_{i}^{\left(j\right)},\;i=1,\ldots ,n$$

where $c_{i}^{\left(j\right)}$ denotes the importance of method *j* at wavelength *i*. This fused curve not only preserves the complementary advantages of multiple methods but also ensures global consistency, smoothness, and rational local structure, thereby providing a robust foundation for subsequent spectroscopic modeling and feature selection.

## 3. Experiments

### 3.1. Experimental Setup

All experiments in this study were conducted on a server equipped with an Intel Core i9-13900K processor (Intel Corporation, Santa Clara, CA, USA) and an NVIDIA GeForce RTX 4090 graphics card (NVIDIA Corporation, Santa Clara, CA, USA). Algorithm development and data analysis were implemented in a Python 3.8 environment, leveraging key machine learning and data processing libraries such as Scikit-learn (v1.0.2), SHAP (v0.44), and Pandas (v1.4). A standardized experimental pipeline was established to ensure the reproducibility of the experimental process and the comparability of results.

### 3.2. Feature Selection Efficacy

An investigation into the role of feature selection reveals its critical importance in enhancing predictive accuracy. As summarized in Table 1, it is clear that using the entire feature set is rarely optimal. Instead, nearly all models achieve lower prediction errors when trained on a carefully selected subset of features. For instance, in the prediction of $M_{ad}$, the Linear Regression model’s MAE improved from 2.666 with the full feature set to a minimum of 1.401 using only the top 20 features. This trend of improved performance with a reduced feature space was consistently observed across most models for both the $M_{ad}$ and $V_{ad}$ targets.

This phenomenon suggests that feature selection effectively eliminates redundant and noisy features, thereby reducing model complexity and enhancing generalization performance. Particularly in scenarios with limited sample sizes, reducing the number of irrelevant or weakly correlated features helps to mitigate overfitting. Therefore, the consistent performance improvements shown by various models across different Top-N feature subsets demonstrate the necessity and effectiveness of feature selection in spectral regression modeling.

### 3.3. Visualization-Based Comparative Analysis

**Figure 6:**
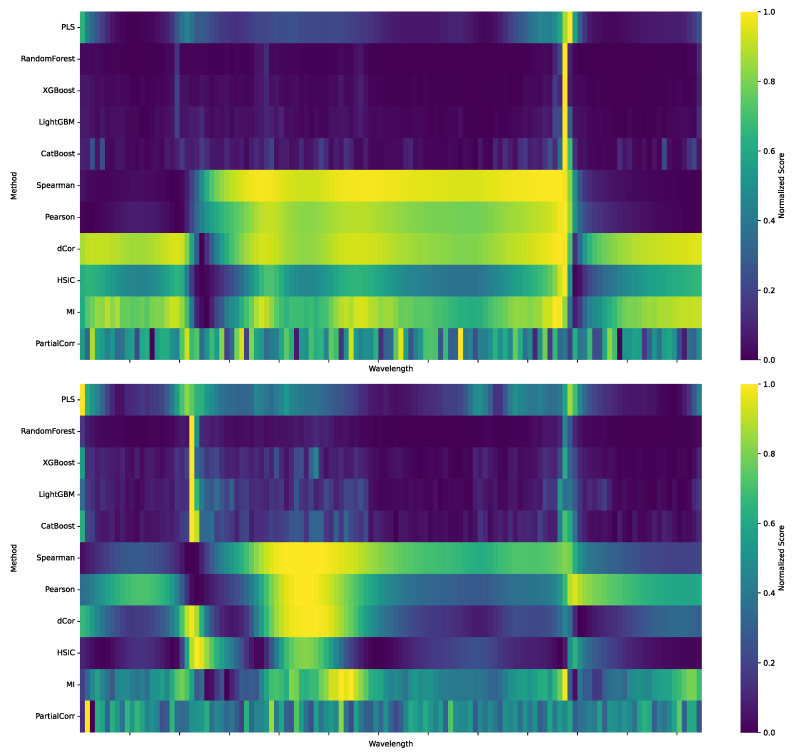
Comparative heatmaps of wavelength importance rankings for predicting $M_{ad}$ (**top**) and $V_{ad}$ (**bottom**) across 11 feature selection methods.

Figure 6 presents the comparative wavelength importance rankings from the eleven feature selection methods. To ensure a fair comparison, all results were normalized. The resulting heatmaps reveal distinct patterns for both $M_{ad}$ and $V_{ad}$. For $M_{ad}$, a strong consensus emerges. Nearly all methods identify the spectral region around 1500 nm as highly important. This indicates that the 1500 nm wavelength range carries a robust and consistent signal across the different methodological families. In contrast, the importance profile for $V_{ad}$ is more dispersed. Rather than being concentrated in one area, high importance is distributed across multiple zones. Several regions were frequently highlighted by the methods, most notably those near 1050 nm and 1500 nm. At the same time, the biases intrinsic to different methodological families are evident. Statistically based approaches such as Pearson [12], Spearman, and dCor [13] tend to yield smooth, broadly distributed profiles, reflecting their reliance on global correlation measures. Model-based approaches such as Random Forest [15], XGBoost [16], and LightGBM [17], by contrast, produce more sparse and sharply peaked profiles, emphasizing localized maxima but with notable volatility across adjacent wavelengths. This contrast illustrates a fundamental methodological trade-off: statistical methods provide stability and smoothness but sacrifice resolution, whereas machine learning methods achieve sharp localization at the expense of stability and reproducibility.

**Figure 7:**
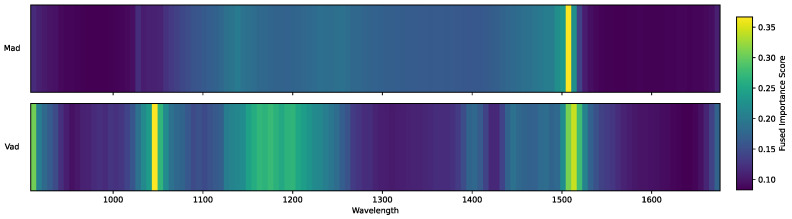
Heatmaps of fused wavelength importance profiles for $M_{ad}$ and $V_{ad}$. The x-axis represents the sequential order of wavelengths, with warmer colors indicating higher relative importance after fusion.

The fused profiles shown in Figure 7 address this challenge by integrating the complementary strengths of different methods. The fusion preserves the consensus trends observed across all statistical measures while incorporating the sharper discriminative signals from machine learning models. As a result, the fused distributions achieve both smoothness and resolution, yielding more coherent and robust profiles than any single method in isolation and highlighting the clear advantage of multi-modal integration.

### 3.4. Spectroscopic and Physicochemical Interpretation

Beyond methodological improvements, the fused profiles allow for direct interpretation in terms of spectroscopic mechanisms and physicochemical relevance. For $M_{ad}$, the most prominent feature is a sharp and narrow peak centered near 1500 nm, corresponding to the first overtone of O–H stretching vibration, a well-established spectral marker of water molecules [26,27,28,29]. Secondary peaks appear near 1140 nm and 1240 nm, arising from combination bands involving O–H stretching and bending vibrations [30], further confirming that the fused method correctly captures the spectral signatures of water content. This is consistent with the fact that $M_{ad}$ is fundamentally determined by moisture, making the O–H vibration bands reliable indicators for its variation.

In contrast, the $V_{ad}$ profile reveals a more dispersed set of high-importance regions around 900 nm, 1040 nm, 1180 nm, and 1510 nm. These bands correspond to overlapping vibrational modes associated with volatile organic matter [4,31]. The absorptions in the 900–1200 nm range can be attributed to overtones and combination bands of C–H stretching vibrations in aliphatic and aromatic hydrocarbons, as well as contributions from C–O and N–H functional groups [32]. The feature near 1040 nm is linked to the second overtone of C–H stretching, while the band around 1180 nm reflects more complex combination modes involving both stretching and bending vibrations [33]. The peak near 1510 nm corresponds to the first overtone of aromatic C–H stretching, consistent with the presence of polyaromatic hydrocarbons and other volatile precursors [28,31]. Unlike the concentrated $M_{ad}$ profile, the broader distribution of $V_{ad}$ importance reflects the inherently heterogeneous nature of volatile matter, which encompasses multiple functional groups and diverse molecular structures.

Taken together, these spectroscopic interpretations confirm that the fused profiles not only achieve methodological robustness but also align closely with known physicochemical mechanisms. By simultaneously enhancing stability, precision, and interpretability, the fusion strategy bridges the gap between purely data-driven feature selection and domain knowledge derived from vibrational spectroscopy, thereby providing a more reliable foundation for both prediction and scientific understanding.

### 3.5. Weighting Analysis of Individual Methods in Fusion

**Figure 8:**
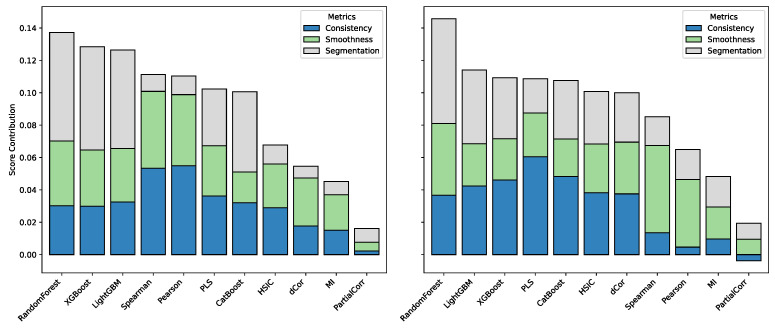
Stacked bar charts of method scores for $M_{ad}$ (**left**) and $V_{ad}$ (**right**). Bar height denotes the total weight of each method, with segments showing contributions from inter-method consistency, smoothness, and local concentration metrics.

To investigate how different feature selection methods contribute to the multi-modal fusion framework, stacked bar charts of method-specific scores were constructed for both $M_{ad}$ and $V_{ad}$, as shown in Figure 8. In these charts, the total bar height represents the overall weight assigned to each method, while the three colored segments correspond to contributions from the inter-method consistency metric, the smoothness metric, and the local segment concentration metric, respectively. This decomposition provides a transparent view of how methodological characteristics are quantitatively reflected in the fusion process.

The results reveal several consistent tendencies. Statistical methods (e.g., Pearson [12], Spearman, and dCor [13]) generally achieve higher scores in the smoothness metric, whereas machine learning–based methods (e.g., Random Forest [15], XGBoost [16], and LightGBM [17]) tend to score higher in the local concentration metric. This distinction aligns well with the visual patterns observed in Figure 6, where statistical approaches emphasize continuous trends across broader regions, while machine learning models capture sharp, localized features. Moreover, Random Forest stands out by obtaining the highest scores simultaneously in both the smoothness and concentration metrics, reflecting its strong capacity to balance broad signal capture with local feature discrimination. By contrast, Partial Correlation (PCor) yields the lowest overall scores across both $M_{ad}$ and $V_{ad}$, consistent with its relatively weak spectral performance.

These findings not only validate the weighting design of the fusion framework but also demonstrate that the derived weights align well with the qualitative spectral behaviors observed in earlier analyses, thereby reinforcing the interpretability and robustness of the proposed integration strategy.

### 3.6. Prediction Performance Evaluation

To quantitatively evaluate the predictive power of the selected wavelength subsets, a rigorous evaluation framework was established. To ensure a robust and generalizable assessment, we employed a 5-fold cross-validation (CV) strategy. This evaluation was performed using a diverse set of regression models, including CatBoost [18], ElasticNet [34], ExtraTrees [35], GradientBoosting [36], KNN, Lasso, LinearRegression, RandomForest [15], Ridge, SVR, and MLP, with the Mean Absolute Error (MAE) adopted as the primary evaluation metric. Specifically, for each feature selection strategy, the prediction errors were averaged over top-N subsets, where N was set to representative values from 50 to 70 at an interval of 5 for both the $M_{ad}$ and $V_{ad}$ prediction tasks. This averaging process ensures a robust and comprehensive assessment of each method’s overall performance.

The detailed results are summarized in Table 2, where the feature selection methods are denoted by abbreviations: CB (CatBoost) [18], HSIC (Hilbert–Schmidt Independence Criterion) [14], LGBM (LightGBM) [17], MI (Mutual Information) [24], PLS (Partial Least Squares), PCor (Partial Correlation), PCC (Pearson Correlation) [12], RF (Random Forest) [15], SRC (Spearman’s Rank Correlation Coefficient), XGB (XGBoost) [16], and dCor (Distance Correlation) [13].

The quantitative analysis reveals the superior performance of the fused method. It secured the top rank in 10 out of 20 model-target combinations, achieving a win rate of 50%. Furthermore, it placed within the top two in 11 out of 20 cases (55%). This success rate is significantly higher than any other single method; for instance, the next best approach (XGB) secured only five top spots, half the number achieved by the fused method. To quantify its overall advantage, the fused method achieved an average reduction of approximately 1.6% in prediction error (MAE) compared to the average performance of the individual feature selection approaches.

**Figure 9:**
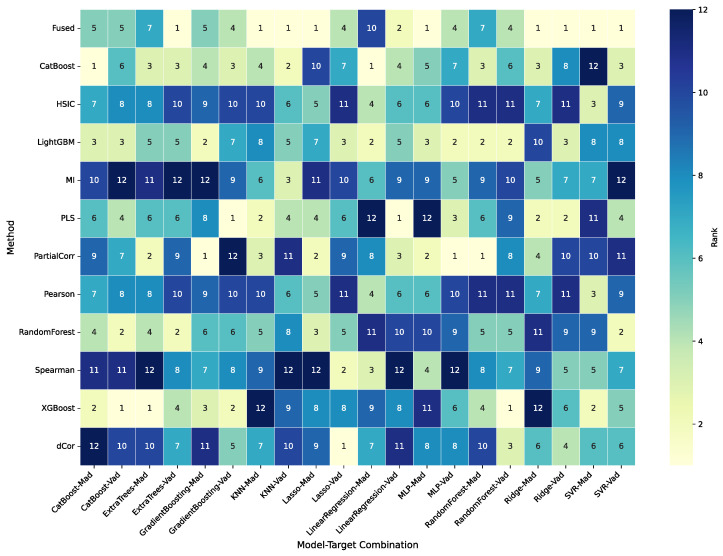
Heatmap of prediction performance rankings (MAE) across different models and feature selection methods.

To further illustrate the method’s consistency, we visualized the ranking distribution in a performance heatmap (Figure 9). The heatmap visually confirms that the fused method not only secured the top rank most frequently but also demonstrated exceptional robustness, as its performance ranking never dropped below tenth place in any scenario. This stability is a key advantage, demonstrating that our fusion strategy avoids drastic performance degradation and maintains balanced effectiveness across heterogeneous modeling scenarios. This overall success stems from the method’s ability to synthesize complementary analytical strengths, integrating the stable, global trends from statistical methods with the sharp, localized power of machine learning models to create a more universally effective feature set.

### 3.7. Stability Analysis

**Figure 10:**
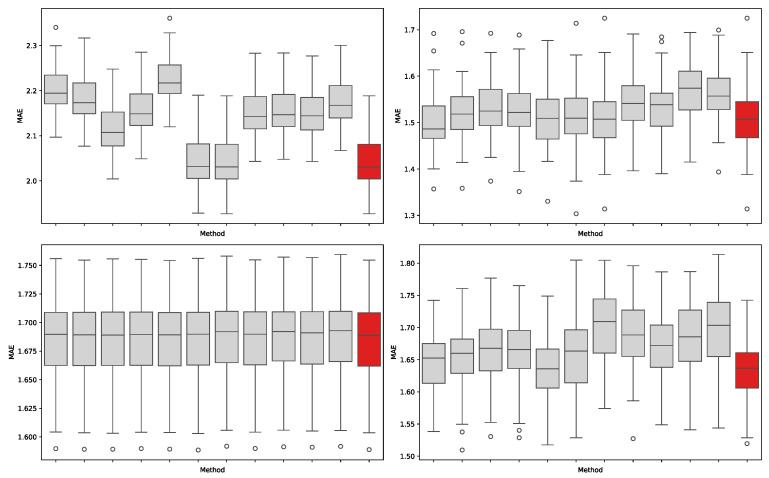
Stability analysis of different feature selection methods under varying random seeds, evaluated by MAE. The four subplots correspond to $M_{ad}$–SVR (**top-left**), $M_{ad}$KNN (**top-right**), $V_{ad}$–SVR (**bottom-left**), and $V_{ad}$KNN (**bottom-right**). Red boxplots represent the fused method, while gray boxplots correspond to individual methods.

As shown in Figure 10, the fused feature selection method exhibits consistently lower and less dispersed error distributions compared to individual methods. This indicates that the fusion approach not only improves accuracy but also enhances stability when the training set is limited. In other words, the fused features provide more reliable predictive performance across varying data partitions, demonstrating strong robustness in small-sample scenarios.

**Figure 11:**
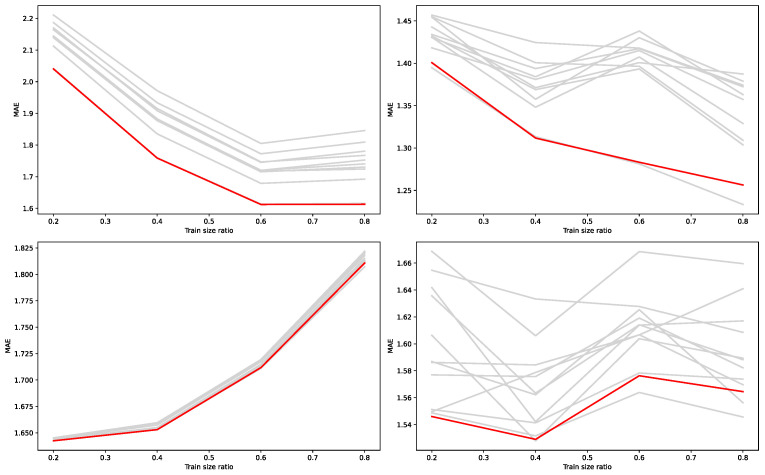
Prediction performance (MAE) of different feature selection methods under varying training set proportions. The four subplots correspond to $M_{ad}$–SVR (**top-left**), $M_{ad}$KNN (**top-right**), $V_{ad}$–SVR (**bottom-left**), and $V_{ad}$KNN (**bottom-right**). Red curves indicate the fused method, while gray curves represent individual methods.

In order to further investigate the effect of training set size on model performance, we evaluated MAE across different train–test ratios for both $M_{ad}$ and $V_{ad}$ using SVR and KNN models. As shown in Figure 11, the fused method consistently outperformed individual methods, with its advantage being especially evident when the training set was limited. These findings further demonstrate the robustness of the proposed fusion strategy under varying data availability.

## 4. Conclusions

In this study, we introduced a novel multi-method analysis framework to address the critical challenge of identifying key spectral features for coal quality assessment with high accuracy and robustness. This framework integrates a diverse range of analytical techniques, including statistical, information-theoretic, machine learning, and chemometric approaches. By doing so, it effectively overcomes the limitations and inherent biases associated with relying on a single method. Our proposed multi-method fusion strategy, based on evaluating inter-method consistency, curve smoothness, and local segment concentration, successfully unified disparate importance profiles into robust representations. The fused profiles clearly demonstrated enhanced interpretability and physicochemical coherence. Quantitatively, our proposed method achieved competitive or superior prediction performance across a wide range of regression models for both $M_{ad}$ and $V_{ad}$. Furthermore, a comprehensive stability analysis confirmed that the fused feature selection method provides significantly more stable and less dispersed error distributions, especially in scenarios with limited training data. In conclusion, the multi-method analysis framework offers a powerful paradigm for spectral feature selection in coal quality assessment. By intelligently integrating complementary analytical perspectives, it leads to more interpretable, accurate, and stable identification of critical wavelengths, thereby providing a robust foundation for optimizing industrial processes and advancing chemometrics.

## Figures and Tables

**Table 1:** MAE of different models with Top-N feature subsets.

	TopN	10	20	40	50	60	70	80	100	All
Model	Target									
CatBoost	$M_{ad}$	1.381	1.335	1.337	1.329	1.325	**1.321**	1.326	1.331	1.343
	$V_{ad}$	1.538	1.545	1.527	**1.525**	1.529	1.530	1.535	1.539	1.537
ExtraTrees	$M_{ad}$	1.391	1.321	1.319	1.311	1.311	1.306	**1.297**	1.307	1.314
	$V_{ad}$	1.524	1.538	1.508	**1.506**	1.507	1.510	1.511	1.515	1.518
GradientBoosting	$M_{ad}$	1.456	1.395	1.393	1.383	1.384	1.377	**1.366**	1.369	1.371
	$V_{ad}$	1.631	1.637	1.603	1.595	1.600	1.598	1.598	1.598	**1.594**
KNN	$M_{ad}$	1.434	**1.429**	1.465	1.480	1.509	1.513	1.513	1.530	1.545
	$V_{ad}$	1.636	1.638	1.630	**1.623**	1.628	1.636	1.650	1.673	1.689
Lasso	$M_{ad}$	1.747	1.731	1.710	1.704	1.694	**1.694**	1.728	1.713	1.713
	$V_{ad}$	1.673	1.673	1.673	**1.667**	1.667	1.668	1.668	1.669	1.669
LinearRegression	$M_{ad}$	1.443	**1.401**	1.435	1.465	1.457	1.521	1.636	1.857	2.666
	$V_{ad}$	1.716	1.804	1.821	**1.617**	1.672	1.767	1.875	2.186	3.303
MLP	$M_{ad}$	**2.713**	2.759	2.759	2.741	2.750	2.779	2.778	2.766	2.759
	$V_{ad}$	**1.924**	2.273	2.877	2.857	3.343	3.563	2.991	3.529	3.938
RandomForest	$M_{ad}$	1.377	1.337	1.336	1.334	1.332	1.332	**1.324**	1.332	1.332
	$V_{ad}$	1.532	1.532	1.519	**1.517**	1.520	1.521	1.521	1.522	1.521
Ridge	$M_{ad}$	2.180	1.850	1.752	1.749	1.752	1.752	1.746	1.723	**1.719**
	$V_{ad}$	1.662	1.661	1.661	1.648	1.648	1.647	**1.647**	1.649	1.650
SVR	$M_{ad}$	2.340	2.266	2.168	2.117	**2.027**	2.043	2.127	2.139	2.179
	$V_{ad}$	1.680	**1.678**	1.680	1.683	1.684	1.684	1.685	1.686	1.687

Notes: **Bold** indicates the best performance in a row. Underline indicate the second best.

**Table 2:** Average Prediction Errors (MAE) of Different Feature Selection Methods Across Regression Models.

		Method											
Model	Target	Fused	CB	HSIC	LGBM	MI	PLS	PCor	PCC	RF	SRC	XGB	dCor
CatBoost	$M_{ad}$	1.140	**1.133**	1.146	1.136	1.150	1.146	1.149	1.146	1.139	1.154	**1.133**	1.154
	$V_{ad}$	1.319	1.320	1.340	1.317	1.372	1.318	1.335	1.340	1.317	1.344	**1.315**	1.341
ExtraTrees	$M_{ad}$	1.100	1.088	1.110	1.089	1.112	1.094	1.084	1.110	1.089	1.113	**1.083**	1.111
	$V_{ad}$	**1.261**	1.262	1.296	1.263	1.309	1.271	1.290	1.296	**1.261**	1.287	1.263	1.286
GradientBoosting	$M_{ad}$	1.191	1.184	1.199	1.182	1.213	1.198	**1.170**	1.199	1.192	1.193	1.183	1.201
	$V_{ad}$	1.376	1.374	1.392	1.379	1.388	**1.369**	1.393	1.392	1.378	1.381	**1.369**	1.377
KNN	$M_{ad}$	**1.292**	1.380	1.412	1.401	1.393	1.357	1.372	1.412	1.391	1.402	1.421	1.400
	$V_{ad}$	**1.510**	1.513	1.534	1.532	1.518	1.531	1.565	1.534	1.536	1.565	1.551	1.564
Lasso	$M_{ad}$	**1.699**	1.732	1.729	1.730	1.733	1.727	1.726	1.729	1.727	1.739	1.731	1.732
	$V_{ad}$	1.624	1.626	1.631	1.623	1.629	1.625	1.628	1.631	1.625	1.622	1.627	**1.620**
LinearRegression	$M_{ad}$	1.105	**1.068**	1.084	1.077	1.084	1.156	1.090	1.084	1.119	1.081	1.091	1.087
	$V_{ad}$	1.280	1.323	1.335	1.325	1.393	**1.273**	1.322	1.335	1.413	1.518	1.342	1.514
MLP	$M_{ad}$	**1.489**	1.532	1.537	1.531	1.546	1.553	1.517	1.537	1.547	1.532	1.553	1.541
	$V_{ad}$	1.625	1.627	1.636	1.623	1.625	1.624	**1.617**	1.636	1.629	1.639	1.627	1.629
RandomForest	$M_{ad}$	1.166	1.161	1.180	1.160	1.176	1.163	**1.130**	1.180	1.163	1.174	1.161	1.177
	$V_{ad}$	**1.330**	1.332	1.353	**1.330**	1.346	1.335	1.335	1.353	1.331	1.333	**1.330**	**1.330**
Ridge	$M_{ad}$	**1.634**	1.655	1.668	1.670	1.667	1.655	1.666	1.668	1.675	1.670	1.682	1.667
	$V_{ad}$	**1.606**	1.613	1.622	1.609	1.612	1.607	1.617	1.622	1.613	1.610	1.612	1.610
SVR	$M_{ad}$	**1.733**	1.881	1.807	1.821	1.819	1.870	1.867	1.807	1.823	1.810	1.806	1.813
	$V_{ad}$	**1.666**	1.668	1.672	1.670	1.674	1.669	1.673	1.672	1.668	1.670	1.669	1.670

Notes: **Bold** indicates the best performance in a row. Underline indicate the second best.

## Data Availability

The data presented in this study are not publicly available due to commercial sensitivity. The Python code and analysis scripts supporting the conclusions of this article are available in the GitHub repository: https://github.com/macabaca/MMISIA (accessed on 17 November 2025).
